# Physical Function and Health‐Related Quality of Life in Adults Treated With Asfotase Alfa for Pediatric‐Onset Hypophosphatasia

**DOI:** 10.1002/jbm4.10395

**Published:** 2020-08-04

**Authors:** Franca Genest, Dominik Rak, Anna Petryk, Lothar Seefried

**Affiliations:** ^1^ Orthopedic Department University of Würzburg Würzburg Germany; ^2^ Alexion Pharmaceuticals, Inc. Boston MA USA

**Keywords:** HYPOPHOSPHATASIA, ENZYME REPLACEMENT THERAPY, PHYSICAL PERFORMANCE, CLINICAL STUDY, REAL‐WORLD EVIDENCE

## Abstract

Hypophosphatasia (HPP) is a rare, inherited, metabolic disease characterized by tissue‐nonspecific alkaline phosphatase deficiency resulting in musculoskeletal and systemic clinical manifestations. This observational study evaluated the effectiveness of enzyme replacement therapy with asfotase alfa on physical function and health‐related quality of life (HRQoL) among adults with pediatric‐onset HPP who received asfotase alfa for 12 months at a single center (ClinicalTrial.gov no.: NCT03418389). Primary outcomes evaluated physical function with the 6‐minute walk test (6MWT), timed up‐and‐go (TUG) test, Short Physical Performance Battery (SPPB), and handheld dynamometry (HHD). Secondary outcome measures included the Lower Extremity Functional Scale (LEFS), pain prevalence/intensity, and pain medication use; HRQoL was evaluated using the 36‐Item Short‐Form Health Survey version 2 (SF‐36v2). Safety data were collected throughout the study. All 14 patients (11 women) had compound heterozygous *ALPL* gene mutations and ≥1 HPP bone manifestation, including history of ≥1 fracture. Mean (min, max) age was 51 (19 to 78) years. From baseline to 12 months of treatment, median 6MWT distance increased from 267 m to 320 m (*n* = 13; *p* = 0.023); median TUG test time improved from 14.4 s to 11.3 s (*n* = 9; *p* = 0.008). Specific components of the SPPB also improved significantly: median 4‐m gait speed increased from 0.8 m/s to 1.1 m/s (*n* = 10; *p* = 0.007) and median repeated chair‐rise time improved from 22 s to 13 s (*n* = 9; *p* = 0.008). LEFS score improved from 24 points to 53 points (*n* = 10; *p* = 0.002). Improvements in HHD were not clinically significant. SF‐36v2 Physical Component Score (PCS) improved after 12 months of treatment (*n* = 9; *p* = 0.010). Pain level did not change significantly from baseline to 12 months of treatment. There were significant improvements on chair‐rise time and SF‐36v2 PCS by 3 months, and on TUG test time after 6 months. No new safety signals were identified. These results show the real‐world effectiveness of asfotase alfa in improving physical functioning and HRQoL in adults with pediatric‐onset HPP. © 2020 The Authors. *JBMR Plus* published by Wiley Periodicals LLC on behalf of American Society for Bone and Mineral Research.

## Introduction

Hypophosphatasia (HPP) is a rare, inherited, metabolic disease characterized by deficiency of tissue‐nonspecific alkaline phosphatase.^(^ ^1^, ^2^ ^)^ Clinical presentation of the disease includes musculoskeletal and systemic signs and symptoms caused by deficient enzyme activity and accumulation of tissue‐nonspecific alkaline phosphatase substrates.^(^ ^3^, ^4^ ^)^ Among other symptoms, these can manifest as muscle weakness, fatigue, chronic pain, dental abnormalities, and fractures or pseudofractures,^(^ ^4^, ^5^, ^6^ ^)^ as well as neurological symptoms.^(^ ^7^ ^)^ Many of these manifestations can impair physical function and reduce health‐related quality of life (HRQoL), and may have a profound impact on the patient's ability to carry out daily activities.^(^ ^4^, ^5^ ^)^

There are limited data available on burden of illness in adult patients with HPP. A study of 125 patients that assessed disease burden using two HPP symptom‐specific surveys found that HPP in adulthood is associated with a high burden of illness^(^ ^5^ ^)^; furthermore, HRQoL, assessed by the 12‐Item Short‐Form Health Survey version 2 in the same cohort of patients, was found to be broadly and substantially affected.^(^ ^5^ ^)^ The Global HPP Registry also found that HPP is associated with numerous signs and symptoms across all ages, and adult patients with HPP often experience a substantial delay in diagnosis.^(^ ^8^ ^)^

Asfotase alfa is a human recombinant enzyme replacement therapy that promotes mineralization of the skeleton, and is the only approved treatment for pediatric and adult patients with pediatric‐onset HPP (conditions of approval vary between regions). Data from several clinical trials on pediatric patients and one study including adolescent and adult patients with HPP demonstrate the efficacy and safety of asfotase alfa over long‐term treatment.^(^ ^9^, ^10^, ^11^, ^12^, ^13^ ^)^

Limited data pertaining to the use of asfotase alfa in routine clinical practice are available; therefore, this study aimed to evaluate the real‐world effectiveness of asfotase alfa in terms of improving physical function and HRQoL among adults with pediatric‐onset HPP, assessed as per standard of care.

## Participants and Methods

### Study design

This is an observational, retrospective, and prospective single‐center study (ClinicalTrial.gov no.: NCT03418389; Evaluate and Monitor Physical Performance of Adults Treated With Asfotase Alfa for Hypophosphatasia) of adults (aged ≥18 years) diagnosed with pediatric‐onset HPP who had received asfotase alfa treatment during routine clinical practice at the Orthopedic Department of the University of Würzburg, Würzburg, Germany, for at least 12 months.

#### *Study population*

Adults with pediatric‐onset HPP (confirmed by low alkaline phosphatase level and/or mutation of the alkaline phosphatase gene [*ALPL*], and clinical signs and/or symptoms consistent with HPP) who had received asfotase alfa for at least 12 months were included. Patients were recruited into the study between August 2018 and December 2018. Informed consent was provided by all individuals before enrollment in the study. HPP care and asfotase alfa treatment were administered according to routine clinical practice, including continuous prescriptions of physiotherapy and manual therapy analogous to previously established individual requirements. The starting dose of asfotase alfa was determined by the treating physician in line with applicable European Medicines Agency Summary of Product Characteristics and the German label. The recommended dosing regimen for asfotase alfa is a s.c. injection of either 2 mg/kg body weight three times a week, or 1 mg/kg body weight six times a week,^(^ ^14^ ^)^ notwithstanding individual dose adjustments following clinical assessment of risk–benefit.

#### *Data collection*

Data were collected retrospectively from patients' electronic and hard‐copy medical records for a look‐back period corresponding to the time from study enrollment to the earliest documented date of HPP diagnosis (or birth, as applicable) on demographics, age at HPP onset, and HPP‐related manifestations. Data collected both retrospectively and prospectively included clinical symptoms, physical function assessments, HRQoL assessments, adverse events (AEs), and asfotase alfa treatment information. Data collection was based on available information from assessments commonly conducted in this patient group at the investigator's site (ie, no investigations were performed outside the standard of care). Baseline clinical evaluations were typically captured within the 6 months before initiation of enzyme replacement therapy. Follow‐up assessments including data collection were regularly scheduled at 3, 6, and 12 months of treatment.

### Variables

#### *Physical function*

Physical function of the patients was assessed using validated qualitative and quantitative measures. Primary outcome measures were the 6‐minute walk test (6MWT),^(^ ^15^ ^)^ timed up‐and‐go^(^ ^16^ ^)^ (TUG) test, Short Physical Performance Battery^(^ ^17^ ^)^ (SPPB; a summary performance measure consisting of a balance test, 4‐m usual gait speed test, and a repeated chair‐rise test), and handheld dynamometry (grip strength) test. The Lower Extremity Functional Scale (LEFS)^(^ ^18^ ^)^ was a secondary outcome measure of physical function. If applicable, it was documented whether the patient required an assistive device (eg, crutches or a walker) to complete these assessments. See the Supplementary Information for further details of each assessment.

#### *Health‐related quality of life and pain*

HRQoL was assessed as a secondary outcome using the 36‐Item Short‐Form Health Survey version 2 (SF‐36v2).^(^ ^19^, ^20^ ^)^ Prevalence of pain was assessed using the following five categories: Never, Rarely, Sometimes, Frequently, and Persistently. If pain was present, pain intensity was quantitated using a 10‐item Likert scale.^(^ ^21^ ^)^ Information on patients' use of pain medication was also collected. See the Supplementary Information for more information on these measures.

#### *Injection‐site reactions and safety reporting*

All patients were advised to continuously monitor injection sites and document any local or systemic observations they may have made themselves in association with the injections.

All serious and nonserious AEs reported by the patient and/or identified in response to open‐ended questions from the treating physician or revealed by observation, physical examination, or other study procedures were documented in the patient's study records/source documents, in accordance with normal clinical practice.

### Data analysis

The SPSS Statistics 25 (SPSS Inc., Chicago, IL, USA) statistical software was used for the data analyses. Quantitative variables were presented as median with interquartile range (IQR) or mean with SD. They were tested for normal distribution using the Shapiro–Wilk test. Owing to significant deviations from normal distribution further analyses were performed using nonparametric methods. Comparisons between baseline and 3, 6, and 12 months of treatment for each variable were assessed using the Wilcoxon matched pairs test; only patients who had data for both of the time points being compared were included in the statistical analyses. For qualitative variables, absolute and percentage frequencies were given. All tests were two‐sided with a significance level of 5%. Owing to the descriptive nature of the present analysis, no alpha adjustment for multiple testing was applied, and the results were interpreted accordingly. Representation of longitudinal results included only those patients with available data for all four time points.

## Results

### Patient demographics and baseline clinical characteristics

Fourteen patients were included in the analysis (11 women, 3 men; their mean age was 51(SD 17) years, ranging from 19 to 78 years (Table 1). All patients were unrelated and had compound heterozygous *ALPL* mutations (Supplementary Table S1) and a documented bone manifestation of the disease (including a history of at least one fracture) in addition to other HPP‐related manifestations. Twelve patients were initiated on an asfotase alfa dosage of approximately 6.0 mg/kg/week; 2 patients who were concerned about potential side‐effects were initiated on 3.0 mg/kg/week. In all patients, the weekly dose was administered on three injection days. Dosing was adjusted as deemed clinically necessary during the 12 months of treatment.

**Table 1:** Patient Demographics and Baseline Clinical Characteristics

Characteristic	Patients (*N* = 14)
Sex, *n* (%)	
Women	11 (79)
Age at baseline, years	
Mean (SD)	51 (17)
Range	19–78
Mean height, cm (SD)	
Men	175 (5)
Women	157 (9)
Mean weight, kg (SD)	
Men	98 (18)
Women	73 (22)
Mean BMI, kg/m^2^ (SD)	
Men	32 (7)
Women	30 (8)
Compound heterozygous mutations of *ALPL*, *n* (%)	14 (100)
Mean ALP activity level at baseline, IU/L (SD)^a^	
Men	14 (4)
Women	18 (7)
Mean PLP activity at baseline, ng/mL (SD)^b^	451 (390)
Mean PEA/creatinine ratio at baseline, mmol/mol creatinine^c^	76 (59)
Menopause status (women only)	
Postmenopausal (*n*, %)^d^	5 (45)
History of HPP‐related manifestations, *n* (%)^e^	
Dental	14 (100)
Fractures	14 (100)
Muscular	14 (100)
Neurological	6 (43)
Renal	3 (21)
Rheumatic	3 (21)
Skeletal^f^	14 (100)
Pain	14 (100)

ALP = alkaline phosphatase; *ALPL* = alkaline phosphatase gene; HPP = hypophosphatasia; PEA = phosphoethanolamine; PLP = pyridoxal 5′‐phosphate.

^a^ Reference ranges for ALP: 53 to 128 IU/L (men), 42 to 98 IU/L (women).

^b^ Reference range for PLP: 5 to 30 ng/mL.

^c^ Reference range for PEA/creatinine ratio: 2.3 to 11.3 mmol/mol creatinine; data were unavailable for 3 patients.

^d^ Calculated as proportion of female patients; data were unavailable for one patient.

^e^ Calculated as proportion of patients for whom data were available.

^f^ Excluding fractures. Skeletal diagnoses included bowing of the long bones, congenital club foot, craniosynostosis, scoliosis, and kyphosis.

### Physical function

#### *Six‐minute walk test*

**Fig 1:**
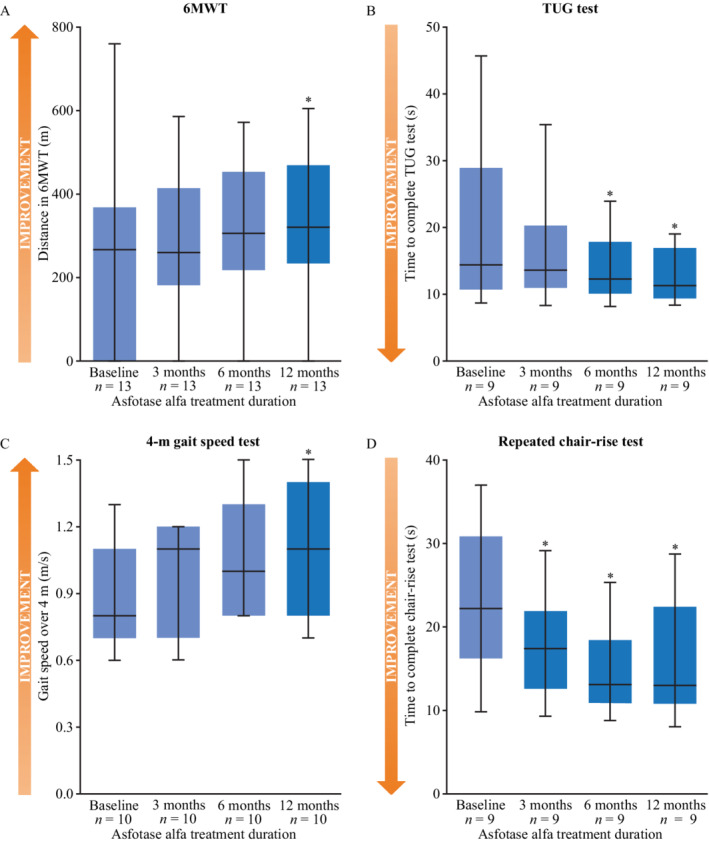
Primary outcomes of physical function as assessed by the (*A*) 6MWT distance, (*B*) TUG test time, (*C*) 4‐m gait speed test, and (*D*) repeated chair‐rise test, among adults treated with asfotase alfa for pediatric‐onset HPP at baseline, 3, 6, and 12 months of treatment. *p < 0.05 versus baseline. The lower and upper boundaries of blue boxes represent the 25th and 75th percentiles, respectively. The horizontal black lines represent the median, and whiskers represent the maximum and minimum values. 6MWT (*n* = 13); TUG test (*n* = 9); 4‐m gait speed (*n* = 10); repeated chair‐rise (*n* = 9). 6MWT = 6‐minute walk test; HPP = hypophosphatasia; SPPB = Short Physical Performance Battery; TUG = timed up‐and‐go.

Of the 14 patients in this study, 13 completed 6MWT assessments at each time point. At baseline, the median distance walked was 267 (IQR, 0 to 368) m (mean 237 m, SD 223 m), which increased to 320 (IQR, 234 to 469) m at 12 months of treatment (mean 335 m, SD 159 m; Fig. 1A). The change in median 6MWT distance walked between baseline and 12 months was 53 m (*p* = 0.023), which corresponded to a 20% improvement. The median changes between baseline and 3 months and between baseline and 6 months of treatment were −7 m and 39 m, respectively.

Seven of the evaluable patients required assistive devices to complete the 6MWT at baseline (3 patients used crutches; 4 used a rolling walker). Two of these patients were able to complete the test unassisted later during the course of the study; one patient was able to complete the test unassisted from 3 months onwards, and another patient was able to complete the 12‐month assessment without assistive devices. None of the patients who walked unassisted at baseline required assistance at any point during the study.

#### *Timed up‐and‐go test*

Nine patients were able to perform the TUG test at all time points. The median time to complete the TUG test was 14.4 (IQR, 10.7 to 28.9) s at baseline and decreased to 11.3 (IQR, 9.4 to 16.9) s after 12 months of treatment (Fig. 1B). The change in median time to complete the test between baseline and 12 months of treatment corresponded to a 22% improvement, and was statistically significant (*p* = 0.008). Median (IQR) time to complete the test at 3 months and 6 months of treatment was 13.6 (IQR, 10.9 to 20.3) s and 12.2 (IQR, 10.1 to 17.8) s, respectively; this change was statistically significant when comparing baseline and 6 months of treatment (*p* = 0.021). One patient required an assistive device to complete this test at baseline, but not at 3 months of treatment.

#### *Short Physical Performance Battery*

Ten patients completed the 4‐m gait speed test, semi‐tandem stand, and tandem stand components of the SPPB at baseline and at follow‐up visits; 9 patients completed the repeated chair‐rise test at all time points. Median speed to walk 4 m was 0.8 (IQR, 0.7 to 1.1) m/s at baseline and 1.1 (IQR, 0.8 to 1.4) m/s at 12 months of treatment (Fig. 1C). The change in median usual gait speed was significant when comparing baseline with 12 months of treatment (*p* = 0.007), and corresponded to a 38% improvement. At 3 months and 6 months of treatment, median gait speed was 1.1 (IQR, 0.7 to 1.2) m/s and 1.0 (IQR, 0.8 to 1.3) m/s, respectively. Four patients required an assistive device to complete this assessment at baseline; 2 of these completed the task unassisted as of 3 months of treatment, and another patient no longer required an assistive device to complete the test after 6 months of treatment.

Median time to complete the repeated chair‐rise test was 22.2 (IQR, 16.2 to 30.8) s at baseline and 13.0 (IQR, 10.8 to 22.4) s at 12 months of treatment (Fig. 1D). Changes compared with baseline were significant at 3, 6, and 12 months of treatment (all *p* = 0.008), and corresponded to a 41% improvement between baseline and 12 months of treatment.

At baseline, all patients were able to hold the semi‐tandem stand for 10 s. One patient was not able to complete this task at 3 months of treatment, but was able to do so at 6 months and 12 months of treatment.

At baseline, 8 patients were able to hold the tandem stand for 10 s. The 2 patients who were not able to complete the task were able to do so after 12 months of treatment. One patient who was able to complete the task at baseline was unable to do so after 12 months of treatment.

#### *Grip strength (dominant hand)*

Grip‐strength measurements were available for 12 of the 14 patients at all time points. Median grip strength was 22.7 (IQR, 11.6 to 25.3) kg at baseline and 22.5 (IQR, 14.3 to 25.4) kg at 12 months of treatment. At 3 months and 6 months of treatment median grip strength was 22.8 (IQR, 12.4 to 25.1) kg and 22.7 (IQR, 13.6 to 25.7) kg, respectively. Although changes were significant at 6 months (*p* = 0.031) and 12 months of treatment (*p* = 0.046) compared with baseline, the change corresponded to just a 1% improvement. The effect size was small, and changes are therefore unlikely to be clinically significant.

#### *Lower Extremity Functional Scale*

**Fig 2:**
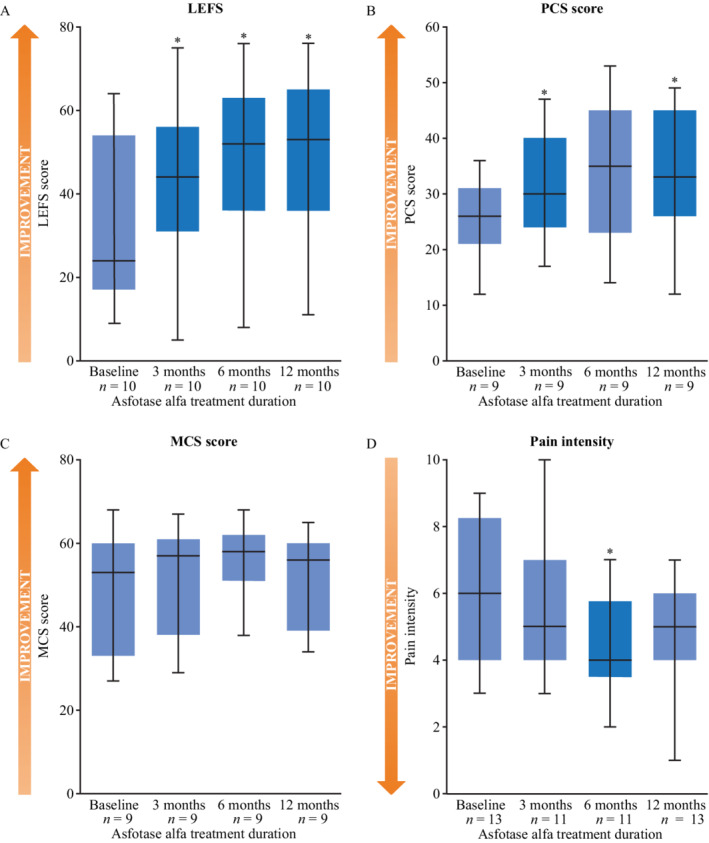
Secondary outcome measures of patient‐reported physical function and burden of disease as assessed by the (*A*) LEFS, (*B*,*C*) SF‐36v2, and (*D*) pain‐intensity questionnaire among adults with pediatric‐onset HPP treated with asfotase alfa for pediatric‐onset HPP at baseline, 3, 6, and 12 months of treatment. *p < 0.05 versus baseline. The lower and upper boundaries of blue boxes represent the 25th and 75th percentiles, respectively. The horizontal black lines represent the median, and whiskers represent the maximum and minimum values. LEFS score (*n* = 10), PCS (*n* = 9), MCS (*n* = 9), pain‐intensity questionnaire: baseline (*n* = 13), 3 months (*n* = 11), 6 months (*n* = 11), 12 months (*n* = 13). HPP = hypophosphatasia; LEFS = Lower Extremity Functional Scale; MCS = Mental Component Summary; PCS = Physical Component Summary; SF‐36v2 = 36‐Item Short‐Form Health Survey version 2.

Ten of the 14 patients included in this study had LEFS data for all time points. At baseline, median LEFS score was 24 (IQR, 17 to 54) points, which increased to 53 (IQR, 36 to 65) points at 12 months of treatment (Fig. 2A). The change in score between baseline and 12 months of treatment was significant (*p* = 0.002) and corresponded to a 121% improvement, which indicates an improvement in the ability to carry out day‐to‐day activities. Median LEFS score was 44 (IQR, 31 to 56) points at 3 months of treatment and 52 (IQR, 36 to 63) points at 6 months of treatment (*p* = 0.009 and *p* = 0.010, respectively, compared with baseline).

### Health‐related quality of life and pain

#### *The 36‐Item Short‐Form Health Survey version 2*

Nine patients completed the SF‐36v2 at all four time points. At baseline, the median Physical Component Summary (PCS) score was 26 (IQR, 21 to 31), which increased to 33 (IQR, 26 to 45) at 12 months of treatment (*p* = 0.010; Fig. 2B); a 27% improvement. Changes were also significant between baseline and 3 months (*p* = 0.028). The median Mental Component Summary (MCS) score was 53 (IQR, 33 to 60) at baseline, and 56 (IQR, 39 to 60) at 12 months of treatment (Fig. 2C); an improvement of 5%. No statistically significant changes were observed at any of the time points compared with baseline for the MCS score.

#### *Pain*

Information on categorical prevalence of pain (categorized as Never, Rarely, Sometimes, Frequently, or Persistently) was available for all 14 patients at baseline, for 12 patients at 3 months and 6 months, and for 13 patients at 12 months. Except for one patient at baseline, all patients reported to be affected by pain at any given time point. At baseline, 9 of 14 patients (64%) reported experiencing persistent or frequent pain; this proportion decreased to 3 of 12 patients (25%) at 6 months, and 5 of 13 patients (38%) at 12 months. Data on pain intensity were available for 13 patients at baseline, for 11 patients at 3 months and 6 months, and for 13 patients at 12 months; if pain was present, its intensity was quantitated using a 10‐item Likert scale (1 = minimal pain, 10 = maximum possible pain). Median pain intensity at baseline was 6 (IQR, 4 to 8.25) points, which decreased to 5 (IQR, 4 to 6) points at 12 months of treatment (Fig. 2D); a 17% improvement. At 3 months and 6 months of treatment median pain intensity was 5 (IQR, 4 to 7) points and 4 (IQR, 3.5 to 5.75) points, respectively. Changes in median pain intensity from baseline to 3 months and 12 months of treatment were not statistically significant; however, a significant decrease in pain intensity compared with baseline was observed at 6 months of treatment (*p* = 0.036).

Twelve patients had pain medication data available at baseline; all of them were using pain medication before initiating asfotase alfa treatment. Eight of these patients used pain medications daily, and 6 patients used a combination of pain medications.

At 6 months of treatment, 2 of these patients were able to discontinue use of pain medication; one of these patients was not using pain medication at 12 months. Over the course of the study, 4 patients reduced their use of pain medication from daily use to an on‐demand basis.

### Injection‐site reactions and safety reporting

Retrospective assessment of available patient records and longitudinal photo documentation of injection sites and injection‐site reactions showed that half of the patients (*n* = 7) self‐administered asfotase alfa injections, and half had their partner or a friend administer the drug.

Although patients were advised to rotate their use of four s.c. injection sites including abdomen, thigh, upper arm, and gluteal area, only one patient continually used all injection‐sites. Eight patients used three different sites; 3 patients used two sites; and 2 patients tolerated injections only at a single injection site (one used the abdomen, one used the gluteal area).

Eleven of the 14 patients noted reddening and/or tenderness at injection sites with variable intensity and duration sometime during the first 3 months of treatment; this increased to 13 patients by 12 months of treatment. Affected injection sites were the abdomen (*n* = 12), thighs (*n* = 4), and upper arm (*n* = 3). Comparing available photographs over the course of the study revealed that 5 patients exhibited faint initial signs of soft tissue distension during the first 3 months of treatment, including bulging of s.c. fat tissue suggesting lipohypertrophy; upon palpation, no bulky fat masses were identified, but rather sagging of the skin suggesting dystrophy of the s.c. fat tissue, providing insufficient suspension for overlying skin. Of the 11 women included in this study, such alterations were visible at the abdomen in 9 of these patients at 12 months of treatment; all of these patients had extensive abdominal fat tissue before treatment. None of these soft tissue distensions receded over time; nevertheless, these findings did not lead to treatment interruption or termination. No tissue distension was observed at any injection site of the two women who were not obese (one of whom did not inject in the abdomen). No relevant tissue distension was seen in men, even though two of them had extensive abdominal fat tissue.

In addition to injection‐site reactions, 46 AEs were recorded in the patients being treated with asfotase alfa for 12 months; all patients experienced at least one AE. The majority of these events (*n* = 33) were not, or were unlikely to be, related to asfotase alfa treatment; they were rather associated with underlying disease and/or comorbidities, such as degenerative disease of the spine, lower back pain/lumbago, knee osteoarthritis, myogelosis (muscle tension/stiffness), greater trochanteric pain syndrome, and skin irritation. The 13 AEs reported as possibly related to treatment with asfotase alfa were fatigue (*n* = 2), weight gain (*n* = 2), headache (*n* = 2), back pain, increase in pain, performance loss in daily activities, insufficiency fracture, raised intraocular pressure, small bowel ileus, and skin irritation (*n* = 1 each).

## Discussion

This study of a real‐world cohort suggests that asfotase alfa is effective in improving physical functioning and HRQoL in adults with pediatric‐onset HPP. The study also highlights the relevance of specific assessment tools to evaluate the effectiveness of treatment and monitoring improvements in patients over time.

Assessments of physical function and HRQoL in this study population before initiation of asfotase alfa treatment indicate a high disease burden in adult patients with pediatric‐onset HPP compared with healthy adult populations of a similar age. For example, the mean ± SD 6MWT distance that patients achieved at baseline in this study was considerably less than in a population of 444 healthy adults (237 ± 223 m versus 571 ± 90 m, respectively).^(^ ^22^ ^)^ Similarly, median TUG test time at baseline was longer than that observed in a cohort of healthy adults in their 50s (14.4 s versus 9.9 s, respectively),^(^ ^23^ ^)^ as was median grip strength (22.7 kg in this study at baseline versus 27 kg [women] and 46 kg [men] in a healthy North American and European population of a similar age).^(^ ^24^ ^)^ Median PCS score on the SF‐36v2 test at baseline was also substantially lower than is observed in the general population in Germany (26 versus 49, respectively); however, baseline median MCS scores in these populations were not remarkably different.^(^ ^25^ ^)^

Of the primary outcome measures of physical function (6MWT, TUG test, SPPB, and grip strength), both the 6MWT and the TUG test, as well as two of the three components of the SPPB, showed statistically significant improvements from baseline to sometime during 12 months of treatment, which indicates the effectiveness of asfotase alfa in improving the functional health of adult patients with pediatric‐onset HPP. Minimum clinically important differences for adults with HPP have been established for the 6MWT (31 m)^(^ ^26^ ^)^; in this study, the median 6MWT distance increased by a clinically meaningful 39 m at 6 months and 53 m at 12 months of treatment compared with baseline. Although improvements observed by 6 months were not significant, statistically significant improvements were noted at 12 months. Similarly, usual gait speed increased during treatment, and this improvement reached statistical significance at 12 months of treatment. In addition, improvements in physical function were observed in the TUG test time and the chair‐rise test. Time to perform these tests decreased significantly by as early as 6 months of treatment for the TUG test and 3 months of treatment for the repeated chair‐rise test.

Grip strength has been recommended as a measure of muscle strength in pediatric patients with HPP,^(^ ^27^ ^)^ and has previously been used in studies of pediatric cohorts^(^ ^28^ ^)^; however, evidence of its use as a measure in adult populations is scarce and its usefulness is uncertain. Changes in grip strength observed during the course of treatment in this study were not clinically significant and not as substantial as improvements in hip extensor and hip abductor strength that were reported in a clinical study of patients receiving asfotase alfa.^(^ ^11^ ^)^ Assessing muscle strength in these patients is likely biased by various confounding factors, including pain and bone and joint issues. Therefore, it remains to be determined how best to measure muscular force and performance in these patients.

Statistically significant improvements were also recorded on the LEFS, the secondary outcome measure of physical function in this study. LEFS score increased significantly by as early as 3 months of asfotase alfa treatment and was sustained at all follow‐up time points compared with baseline. These increases were substantially more than the minimum clinically important difference of 9 points calculated by Binkley and colleagues for a population of patients with lower‐extremity musculoskeletal dysfunction,^(^ ^18^ ^)^ and those observed in a clinical trial population of adults receiving asfotase alfa over a similar timeframe,^(^ ^11^ ^)^ and showed an improvement in patients' ability to carry out day‐to‐day tasks.

Improvements in physical well‐being were also reflected by the significant improvements in the secondary outcome measures of HRQoL. Improvements in median SF‐36v2 PCS score during the course of the study were statistically significant at 3 months and 12 months of asfotase alfa treatment compared with baseline. This indicates an overall improvement in physical health, comprehensively assessing domains of physical functioning, physical role functioning, bodily pain, and general health. Although the median MCS score (which encompasses measures of vitality, social functioning, emotional role functioning, and mental health) also increased slightly at all time points compared with baseline, this was not significant. Given that several studies have described the mental and emotional impact of HPP on adult patients,^(^ ^5^ ^)^ the similarity in the MCS scores observed among the general German population and this study cohort at baseline suggests that the SF‐36v2 may not be an accurate measure of mental well‐being in patients with HPP.^(^ ^25^ ^)^

The negative impact of pain on HRQoL is well established.^(^ ^29^ ^)^ All patients included in this study had experienced pain; however, although median pain intensity reported across the cohort decreased significantly by 6 months of asfotase alfa treatment, pain intensity increased again by 12 months of treatment. Given that marked improvements were observed in physical function during this timeframe, it is possible that this may be owing to patients becoming more active during the course of their treatment. A 5‐year study of the efficacy of asfotase alfa in adult and adolescent patients with HPP found that greater reductions in pain levels were observed after 5 years of treatment^(^ ^11^ ^)^; therefore, it is also possible that the follow‐up period in this interim analysis was too short to observe any meaningful decrease in pain. Patient perception of overall pain may also change over time, and this assessment tool may not be sufficiently specific to observe consistent changes in pain associated with HPP in adults.

A similar frequency of AEs and safety events was reported in the current study as observed in clinical trials,^(^ ^9^, ^10^, ^11^, ^12^, ^13^ ^)^ in which asfotase alfa was well tolerated; no new safety signals emerged from this study. Injection‐site reactions appear to be quite common, specifically reddening and tenderness at injection sites. In addition, soft tissue alterations at abdominal injection sites, including lipodystrophy with skin sagging resembling lipohypertrophy, are common and women with extended abdominal fat tissue appear to be prone to develop these.

Together, these results indicate that asfotase alfa is an effective treatment for improving physical functioning and HRQoL of patients with HPP, and demonstrates a favorable safety profile.

Several of the physical function and HRQoL assessments used in this study have been recommended for the treatment monitoring of adult patients with HPP, including the 6MWT and SF‐36v2.^(^ ^27^ ^)^ Results of this study suggest that specific measures of physical function and HRQoL used in this analysis, including the 6MWT, the TUG test, the repeated chair‐rise test of the SPPB, the LEFS, and the PCS component of the SF‐36v2 can measure effectiveness of asfotase alfa, some as early as 3 months after treatment initiation. Of these assessments, the LEFS showed the largest percentage improvement between baseline and 12 months of treatment, indicating that it may be the most suitable scale for evaluating treatment effectiveness with regard to functional performance in this patient population. We recommend that these tests be considered by clinicians as part of routine practice to monitor early treatment response in adults with HPP, especially because they are practical and easy to implement in a clinical environment with minimal technical equipment and training. It remains to be determined to what extent these measurements can also be helpful to monitor long‐term effectiveness of treatment; further research is required to demonstrate the usefulness of these measures when monitoring patients on treatment for more than 12 months.

In addition to the data we present here on validated assessments of physical function and HRQoL, it is worth noting that all 14 patients included in this study had a history of dental abnormalities. This highlights the importance of good dental networks within communities, to ensure that diseases of bone metabolism (including HPP) are recognized early and appropriate follow‐up care can be implemented. Such an approach is especially important, considering that dentists may often be among the first health care providers in the clinical pathway for patients with HPP and other diseases of bone metabolism. Limitations of this study include the small sample size and lack of a comparator arm; however, owing to the rarity of HPP and its debilitating nature, inclusion of a comparator arm (which would involve no treatment) would be unethical and would not be in accordance with the descriptive nature of this study. Additionally, 53% of the patients included in this study had a c.571G>A mutation of the *ALPL* gene. Although diligent assessment of familial history did not reveal any kinship among participants, this could not be ruled out because a haplotyping approach was not applied. Nevertheless, a similar proportion of individuals (55%) was found to carry this mutation in a comparable HPP cohort,^(30^ ^)^ confirming that this mutation is common among patients with HPP of European ancestry. Limitations inherent to the use of real‐world data collected in routine clinical practice, and in particular the introduction of bias owing to missing data, are also relevant for this study. These limitations should be considered when interpreting these results. In addition, the fact that this study covers a limited number of patients from a single center may restrict the generalizability of the results. However, to the best of our knowledge this is currently the largest and most consistently monitored cohort of adult patients with HPP receiving long‐term treatment with asfotase alfa. These results establish a starting point of how to monitor treatment in these patients, specifically what can be expected regarding treatment effectiveness and clinical improvement and what to focus on to reduce AEs.

## Conclusion

These data from a real‐world cohort of adult patients with pediatric‐onset HPP suggest that asfotase alfa can be effective in improving physical functioning and HRQoL. This study also highlights the relevance and suitability of specific practical assessment tools, including the 6MWT, the repeated chair‐rise component of the SPPB, the TUG test, the LEFS, and the PCS component of the SF‐36v2 to evaluate the effectiveness of asfotase alfa and monitor improvements in patients over time.

## Disclosures

FG is a clinical study investigator and has received speaker honoraria from Alexion Pharmaceuticals, Inc. DR has no conflicts of interest to disclose. AP is employed by Alexion and may have stock options/ownership. LS is a clinical study investigator and has received consultancy fees and institutional research funding and/or grant support from Alexion Pharmaceuticals, Inc.

## Author contributions

**Franca Genest**: Data curation; formal analysis; investigation; methodology; validation; writing‐original draft; writing‐review and editing. **Dominik Rak**: Data curation; investigation; methodology; validation; writing‐review and editing. **Anna Petryk**: Conceptualization; methodology; validation; writing‐original draft; writing‐review and editing. **Lothar Seefried**: Conceptualization; methodology; data curation; formal analysis; investigation; validation; writing‐original draft; writing‐review and editing.

### Peer Review

The peer review history for this article is available at https://publons.com/publon/10.1002/jbm4.10395.

## Supporting information


**Supplementary Table S1** Patient genotype
