# Supplementary material for: Physical Function and Health‐Related Quality of Life in Adults Treated With Asfotase Alfa for Pediatric‐Onset Hypophosphatasia
Source: JBMR Plus. 2020 Aug 4;4(9):e10395. doi: 10.1002/jbm4.10395 (PMC7507107; doi:10.1002/jbm4.10395)
Supplement: Supplementary file 1 — Supplementary Table S1 Patient genotype [file JBM4-4-e10395-s001.docx]

# Supplemental Information

## Physical function variables

#### 6-minute walk test

The maximum distance that the patient could walk within 6-minutes was assessed.^(1)^ A 30 m walking course was used; the length of the course was marked in meter intervals, and turnaround points were clearly indicated. The 6-minute walk test (6MWT) had been validated and a specific minimum clinical important difference for children and adults with hypophosphatasia (HPP) had been established as 31 m.^(2)^

#### Timed up-and-go test

The timed up-and-go (TUG) test measured the time taken for the patient to stand up from a standard arm chair, walk a distance of 3 m at a comfortable and safe pace, turn, walk back to the chair and sit down again.^(3)^

#### Short Physical Performance Battery

The Short Physical Performance Battery (SPPB) is a summary performance measure consisting of a balance test, 4-m gait speed test and a repeated chair rise test.^(4)^ For the balance test the patient was required to hold their balance for 10 seconds while standing unassisted in three positions: feet together (side by side, heels touching), semi tandem (heel of one foot touching the big toe of the other foot), and tandem (feet directly in front of the other; the heel of one foot touching the toes of the other foot). The 4 m gait speed test measured the time it took the patient to walk at their usual pace for 4 m, from a standing start; walking speed was calculated from the fastest of three attempts. For the repeated chair-rise test, the time it took for the patient to rise to a fully standing position from a seated position on chair, with the legs at 90˚, five times in succession was recorded. A chair with a straight back and without arm rests was used.

#### Grip strength

Grip strength of the patient’s dominant hand was measured using a hand-held dynamometer while they were sitting in a comfortable position with the elbow flexed to 90˚; a mean of three measurements was recorded.

#### Lower Extremity Functional Scale

The Lower Extremity Functional Scale (LEFS) is a self-reported questionnaire used to assess the patient’s ability to perform 20 everyday tasks.^(5)^ Performing each activity was ranked by the patient as: extreme difficulty or unable to perform activity (score of 0); quite a bit of difficulty (score of 1); moderate difficulty (score of 2); a little bit of difficulty (score of 3); no difficulty (score of 4). The sum of the scores was calculated, up to a possible maximum of 80.

## Health-related quality of life variables

36-Item Short-Form Health Survey version 2
The 36-Item Short-Form Health Survey version 2 (SF-36v2) is a multi-purpose health survey comprised of 36 questions.^(6)^ It consists of eight subscales: physical functioning; physical role functioning; bodily pain; general health perception; vitality; social functioning; emotional role functioning and mental health. The eight subscales are used to generate two component scores: a Physical Component Summary (PCS) score and a Mental Component Summary (MCS) score.

#### Pain

Pain was assessed using an individually developed questionnaire in which patients were asked to quantitate their perception of pain on a 10-item Likert scale.

## Supplementary Table S1. Patient genotype

| **Patient** | **Molecular genetic test** | **Exon** | **cDNA mutation** | **Protein level** |
| --- | --- | --- | --- | --- |
| 21001 | Compound heterozygous mutation | 6  9 | c.571G>A  c.984_986delCTT | p.Glu191Lys p.Phe328del |
| 21002 | Compound heterozygous mutation | 6  6 | c.535G>A  c.571G>A | p.Ala179Thr  p.Glu191Lys |
| 21003 | Compound heterozygous mutation | 6  11 | c.571G>A  c.1250A>G | p.Glu191Lys  p.Asn417Ser |
| 21004 | Compound heterozygous mutation | 5  11 | c.382G>A  c.1276G>A | p.Val128Me  p.Gly426Ser |
| 21005 | Compound heterozygous mutation | 6  10 | c.571G>A  c.1001G>A | p.Glu191Lys  p.Gly334Asp |
| 21006 | Compound heterozygous mutation | 6  10 | c.571G>A  c.1001G>A | p.Glu191Lys  p.Gly334Asp |
| 21007 | Compound heterozygous mutation | 10  12 | c.1018C>T  c.1310C>T | p.His340Tyr  p.Ala437Val |
| 21008 | Compound heterozygous mutation | 6  12 | c.571G>A  c.1354G>A | p.Glu191Lys  p.Glu452Lys |
| 21009 | Compound heterozygous mutation | 6  6 | c.500C>T  c.571G>A | p.Thr167Met  p.Glu191Lys |
| 21010 | Compound heterozygous mutation | 3  7 | c.119C>T  c.746G>T | p.Ala40Val  p.Gly249Val |
| 21011 | Compound heterozygous mutation | 6  10 | c.526G>A  c.1114_1115delCT | p.Ala176Thr  p.Leu372Aspfs*32 |
| 21012 | Compound heterozygous mutation | 6  7 | c.526G>A  c.661G>T | p.Ala176Thr  p.Gly221Cys |
| 21014 | Compound heterozygous mutation | 6  10 | c.571G>A  c.1001G>A | pGlu191Lys  p.Gly.224Asp |
| 21015 | Compound heterozygous mutation | 5  6 | c.379A>G  c.526G>A | p.Thr127Ala  p.Ala176Thr |

## References

1. ATS statement: guidelines for the six-minute walk test. Am J Respir Crit Care Med. 2002;166(1):111–7.

2. Phillips D, Tomazos IC, Moseley S, L'Italien G, Gomes da Silva H, Lerma Lara S. Reliability and validity of the 6-Minute Walk Test in hypophosphatasia. JBMR Plus. 2019;3(6):e10131.

3. Podsiadlo D, Richardson S. The timed "Up & Go": a test of basic functional mobility for frail elderly persons. J Am Geriatr Soc. 1991;39(2):142–8.

4. Guralnik JM, Simonsick EM, Ferrucci L, et al. A short physical performance battery assessing lower extremity function: association with self-reported disability and prediction of mortality and nursing home admission. J Gerontol. 1994;49(2):M85–94.

5. Binkley JM, Stratford PW, Lott SA, Riddle DL. The lower extremity functional scale (LEFS): scale development, measurement properties, and clinical application. Phys Ther. 1999;79(4):371–83.

6. rand.org. 36-item short forum survey (SF-36). Available from: <https://www.rand.org/health-care/surveys_tools/mos/36-item-short-form.html> (Accessed 20 December 2019).
